# Evaluation of clinical outcomes with propensity‐score matching for colorectal cancer presenting as an oncologic emergency

**DOI:** 10.1002/ags3.12557

**Published:** 2022-03-19

**Authors:** Katsuhiro Ogawa, Yuji Miyamoto, Kazuto Harada, Kojiro Eto, Hiroshi Sawayama, Shiro Iwagami, Masaaki Iwatsuki, Yoshifumi Baba, Naoya Yoshida, Hideo Baba

**Affiliations:** ^1^ 13205 Department of Gastroenterological Surgery Graduate School of Life Science Kumamoto University Kumamoto Japan

**Keywords:** bowel obstruction, bowel perforation, colorectal cancer, oncologic emergency, propensity‐score matching

## Abstract

**Aim:**

Oncologic emergencies such as perforation and obstruction associated with colorectal cancer are serious diseases that can lead to sepsis. Peritoneal dissemination and other factors may cause cancer progression and worsen the patients’ long‐term prognosis. In this study, we investigated the effect of colorectal cancer presenting as oncologic emergencies on the patients’ clinical course.

**Methods:**

We performed a retrospective study that included 448 patients with colorectal cancer who underwent primary resection at our institution between January 2014 and December 2018. The primary outcome was overall survival, while secondary outcomes were 30‐day mortality and postoperative complications. Cox regression was used to estimate the hazard ratio (HR) for overall survival.

**Results:**

We identified 56 patients who presented with oncologic emergencies (OE group) and 392 patients who presented with no emergencies (NE group). Propensity‐score matching yielded 56 patients in the OE group and 55 in the NE group with balanced baseline covariates. We found a strong association between overall survival (OS) and oncologic emergencies (HR = 2.4; 95% confidence interval [CI], 1.1‐5.5). The 30‐day mortality was not significantly different between the OE and NE groups (4% vs 0%, *P* = .25). The incidence of severe postoperative complications (Clavien‐Dindo classification ≥grade 3) did not differ significantly between the groups (25% vs 15%, *P* = .23).

**Conclusion:**

Colorectal cancer presenting as an oncologic emergency could be safely operated on without increasing the 30‐day mortality rate and the incidence of severe postoperative complications. However, the long‐term prognosis was poor.

## Graphical abstract

The OS of the OE group was significantly worse than that of the NE group in the total cohort (*P* = .0001; Figure 1a). The OS of the OE group tended to indicate a worse prognosis in the matching cohort (*P* = 0.068; Figure 1b).

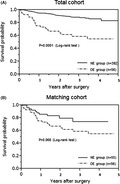

## 1 INTRODUCTION

Colorectal cancer (CRC) is the third most common malignancy in the world and the fourth leading cause of cancer‐related deaths.^1^ Many patients with CRC present with acute or emergent malignancy‐related symptoms. CRC presenting as an oncologic emergency has been reported to occur in 9%‐33% of cases.^2^, ^3^, ^4^

Oncologic emergencies in CRC present with conditions such as bowel perforation, bowel obstruction, and abscess formation. These emergencies in CRC are associated with higher rates of postoperative complications and operative mortality and worse long‐term outcomes in comparison with elective surgery.^5^, ^6^ On the other hand, some reports have described that there are no differences in long‐term prognosis, which is controversial.^7^ The high mortality rate in cancer patients with perforation has been attributed to the cumulative effect of increasing age and debility, sepsis, more advanced malignancy at presentation, preexisting comorbidities, and lower rates of curative resection.^8^

This study aimed to evaluate the short‐ and long‐term outcomes of patients with oncologic emergency for CRC in comparison with a matched patient group without oncologic emergencies by using propensity‐score matching.

## 2 PATIENTS AND METHODS

A total of 448 patients with colorectal cancer who underwent primary tumor resection at Kumamoto University Hospital between January 2014 and December 2018 were included in this study. We compared the oncologic emergency group (OE group) and the non‐emergency group (NE group) and used propensity‐score matching to adjust baseline differences between the groups. This study was approved by the Human Ethics Review Committee of the Graduate School of Medicine, Kumamoto University (ethical approval no.1047).

Oncologic emergency was defined as follows.

1. abscess formation or penetration: patients with intra‐abdominal abscess or findings indicating penetration with ductal organs such as the bladder.
2. bowel perforation: abdominal findings and imaging findings showing perforated peritonitis.
3. bowel obstruction: symptoms of bowel obstruction necessitating hospitalization.

We collected baseline data such as age, sex, body mass index (BMI), and American society of anesthesiologist physical status (ASA‐PS). In addition, we collected data for tumor‐related factors (tumor location and TNM classification) and operation‐related factors (operation time, blood loss, and level of lymph node dissection).

The primary endpoint was overall survival (OS). The secondary endpoints were the 30‐day mortality rate, postoperative complication rate, and hospital stay. Risk factors for OS were also determined by multivariate analysis.

### 2.1 Statistical methods

Variables are shown as median (25%‐75% interquartile range) or number (percentage) of patients. Univariate analyses were performed using the χ^2^ test for categorical variables and the Mann‐Whitney *U*‐test for continuous variables. In all tests, two‐tailed *P*‐values <.05 were considered statistically significant. We used JMP version 10.0.2 (SAS Institute, Cary, NC, USA) for statistical analyses. Propensity‐score matching was performed with EZR (Saitama Medical Center, Jichi Medical University, Saitama, Japan), which is a graphical user interface for R (The R Foundation for Statistical Computing, Vienna, Austria).

The propensity score was calculated by logistic regression for estimating the probability that a patient would show oncologic emergencies. We defined the following variables as potential confounders: age, sex, BMI, ASA‐PS, tumor location, TNM classification, operation time, blood loss, and level of lymph node dissection. Covariates in the model were derived from the list of baseline covariates considered according to clinical expertise. The most common implementation of propensity‐score matching is for one‐to‐one or pair matching, in which pairs of treated and untreated subjects are formed such that matched subjects have similar propensity scores. Caliper was not specified in this study.

## 3 RESULTS

### 3.1 Clinical characteristics of the patients

A total of 448 patients were analyzed during the study period (Table 1). The median patient age was 69 (61‐77) years, 253 patients (57%) were men, and the median ASA‐PS was 2 (2‐2). Fifty‐six patients (13%) presented with oncologic emergencies. Oncologic emergencies included abscess formation or penetration in four cases (1%), bowel perforation in five cases (1%), and bowel obstruction in 49 cases (11%).

**TABLE 1:** Patients' characteristics

Patient characteristics (n = 448)	
Age (years old)	69 (61‐77)
Male	253 (57)
BMI (kg/m^2^)	22 (20‐25)
ASA‐PS	2 (2‐2)
Oncology emergency	56 (13)
Abscess/penetration	5 (1)
Perforation	5 (1)
Bowel obstruction	49 (11)
《Tumor‐related factor》	
Right/left	133/315
Colon/rectum	284/164
Early/advance	93/355
pT3 or pT4	289 (65)
pN+	84 (19)
Synchronous distance metastasis	84 (19)
pStage0/1/2/3/4	16/112/124/105/91
《operation procedure》	
Laparotomy	60 (13)
Stoma	92 (21)
Opreration time (min)	322 (242‐448)
Blood loss (mL)	50 (10‐229)
Lymph node dissection D2 or D3	432 (96)

Abbreviations: ASA‐PS, American Society of Anesthesiologists physical status classification system; BMI, body mass index; PS, performance status.

Detailed information regarding the oncologic emergency group is provided in Table 2. Among the patients who presented with abscess formation or penetration, two underwent emergency surgery and two underwent elective surgery after colostomy. Among the patients showing bowel perforation, four underwent emergency Hartmann's operation and one underwent elective surgery after emergency colostomy. Among the patients with bowel obstruction, 16 had right‐sided colon cancer and 33 had left‐sided colon and rectal cancer. Of the 16 patients with right‐sided colon cancer, nine improved with fasting and administration of fluids and underwent elective surgery. Two patients received transnasal long tubes and one patient received a self‐expandable metallic stent (SEMS), followed by a standby surgery after the bowel obstruction improved. Four patients underwent emergency surgery. Of the patients with left‐sided colon and rectal cancer, 16 patients improved with fasting and administration of fluids and underwent elective surgery. Nine patients underwent insertion of transanal long tubes and five patients received SEMSs, followed by standby surgery when the bowel obstruction improved. Three patients underwent emergency surgery. Table 3 compares the background characteristics of patients in the OE group (56 patients) and the NE group (392 patients). No significant differences were observed in age, sex, BMI, or ASA‐PS score between the two groups. Tumor‐related factors such as pT3/T4 (98% vs 60%; *P* = .0001), pN+ (59% vs 37%; *P* = .002), and synchronous distant metastasis (43% vs 15%; *P* = .0001) were significantly more advanced in the OE group. Among surgery‐related factors, the rate of laparotomy tended to be higher, and the operation time tended to be shorter in the OE group. The level of lymph node dissection was significantly lower in the OE group.

**TABLE 2:** Details of the oncologic emergency group

Details of the oncologic emergency group	
Oncology emergency	
Abscess/penetration	n = 4
Emergency primary tumor resection	2 (50)
Elective surgery after emergency colostomy	2 (50)
Perforation	n = 5
Emergency Hartmann's operation	4 (80)
Elective surgery after emergency colostomy	1 (20)
Bowel obstruction	n = 49
Elective surgery after fasting and administration of fluids	25 (51)
Elective surgery after decompression with the long tube	11 (22)
Elective surgery after decompression with SEMS	6 (12)
Emergency primary tumor resection	2 (4)

Abbreviations: SEMS, self‐expandable metallic stent.

**TABLE 3:** Comparison of patients' characteristics between the two groups before propensity‐score matching

	OE group (n = 56)	NE group (n = 392)	*P* value
Age (years old)	68 (64‐76)	69 (61‐77)	.73
Male	30 (54)	223 (57)	.667
BMI (kg/m^2^)	22 (20‐24)	23 (20‐25)	.1
ASA‐PS	2 (2‐2)	2 (2‐2)	.31
《Tumor‐related factor》			
Right/left	18/38	115/277	.64
Colon/rectum	43/13	241/151	.026
Early/advance	1/55	92/300	.0001
pT3 or pT4	55 (98)	234 (60)	.0001
pN+	33 (59)	145 (37)	.0021
Synchronous distance metastasis	24 (43)	60 (15)	.0001
pStage0/1/2/3/4	0/0/17/15/24	16/112/107/90/67	.0001
《Surgery‐related factor》			
Laparotomy	12 (21)	48 (12)	.089
Stoma	12 (21)	80 (20)	.86
Operation time (min)	314 (227‐387)	325 (247‐452)	.086
Blood loss (mL)	98 (10‐424)	49 (10‐212)	.277
Lymph node dissection D2 or D3	50 (89)	382 (97)	.0087

Abbreviations: ASA‐PS, American Society of Anesthesiologists physical status classification system; BMI, body mass index; PS, performance status.

Propensity‐score matching yielded 56 patients in the OE group and 55 in the NE group with balanced baseline covariates (Table 4). These groups no longer showed significant differences for the tumor and surgery‐related factors that were significantly different before matching.

**TABLE 4:** Comparison of patients' characteristics between the two groups after propensity‐score matching

	OE group (n = 56)	NE group (n = 55)	*P* value
Age (years old)	68 (64‐76)	72 (64‐80)	0.07
Male	30 (54)	28 (51)	0.85
BMI (kg/m^2^)	22 (20‐24)	22 (19‐24)	0.92
ASA‐PS	2 (2‐2)	2 (2‐2)	0.58
《Tumor‐related factor》			
Right/left	18/38	18/37	1
Colon/rectum	43/13	42/13	1
Early/advance	55 (98)	53 (96)	0.61
pT3 or pT4	55 (98)	53 (96)	0.618
pN+	33 (59)	36 (65)	0.558
Synchronous distance metastasis	24 (43)	23 (42)	1
pStage0/1/2/3/4	0/0/17/15/24	1/1/15/15/23	0.71
《Surgery‐related factor》			
Laparotomy	12 (21)	12 (22)	1
Stoma	12 (21)	14 (25)	0.86
Operation time (min)	314 (227‐387)	300 (229‐423)	0.84
Blood loss (ml)	98 (10‐424)	35 (10‐191)	0.24
Lymph node dissection D2 or D3	50 (89)	51 (93)	0.91

Abbreviations: ASA‐PS, American Society of Anesthesiologists physical status classification system; BMI, body mass index; PS, performance status.

### 3.2 Primary outcome

**FIGURE 1:**
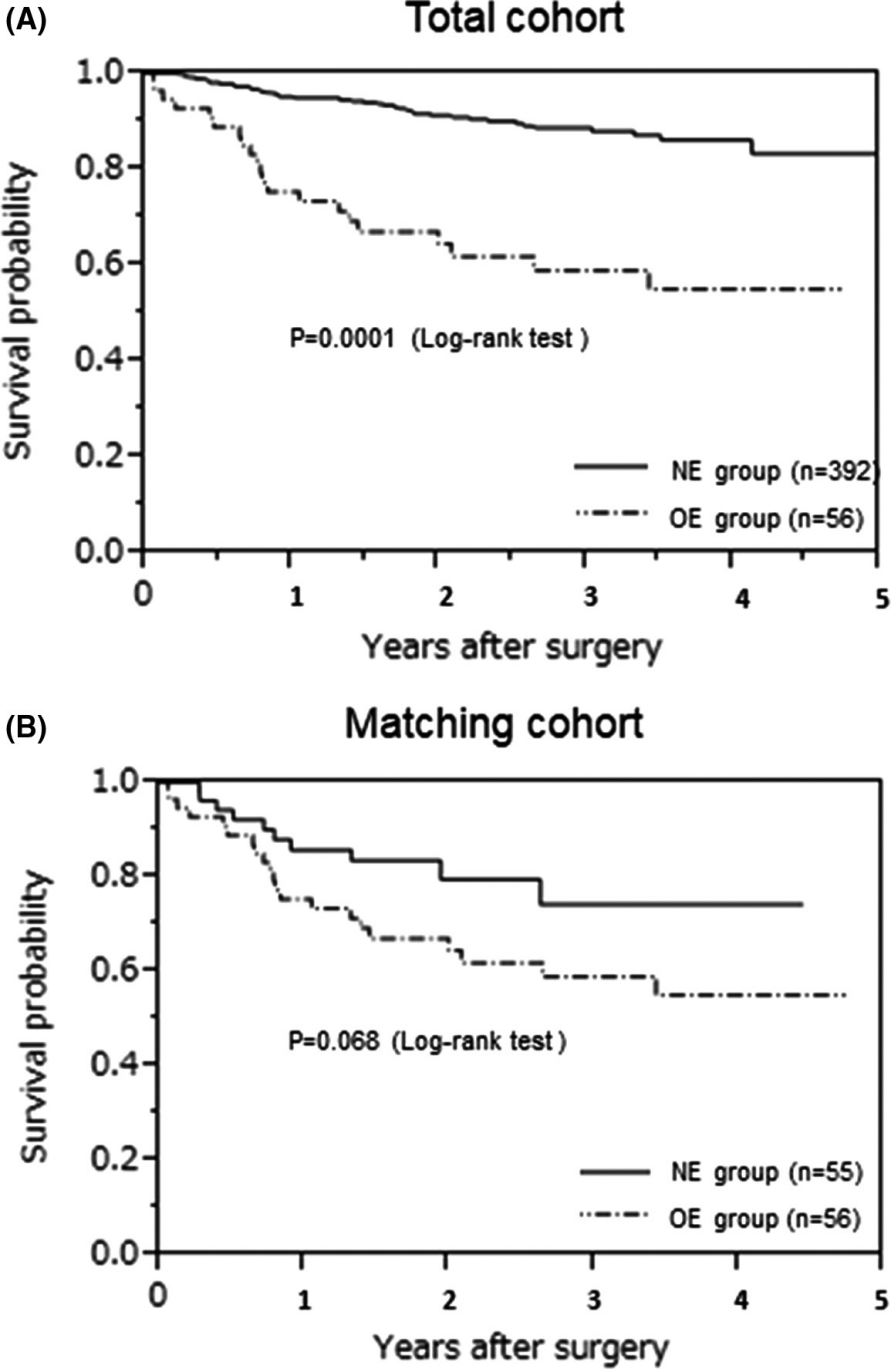
Kaplan‐Meier curve for overall survival. (A) Total cohort, (B) matching cohort

The OS of the OE group was significantly worse than that of the NE group in the total cohort (*P* = .0001; Figure 1A). The OS of the OE group tended to indicate a worse prognosis in the matching cohort (*P* = .068; Figure 1B).

### 3.3 Secondary outcomes

Secondary outcomes are shown in Table 5. The 30‐day mortality showed no significant difference between the two groups (4% vs 0%; *P* = .25), and there was no significant difference in the incidence of serious postoperative complications (Clavien‐Dindo classification ≥3) between the two groups (25% vs 15%; *P* = .23). The duration of hospitalization was significantly longer in the OE group (25 vs 16 days; *P* = .0014). The length of postoperative hospital stay did not differ between the groups, although the length of preoperative hospital stay was significantly longer in the OE group (8 vs 2 days; *P* = .0001).

**TABLE 5:** Short‐term outcomes between the two groups

	OE group (n = 56)	NE group (n = 55)	*P* value
30‐day mortality	2 (4)	0 (0)	0.25
Total SSI rate	10 (18)	9 (16)	1
Superficial and deep SSI	1 (2)	2 (4)	0.61
Organ and space SSI	9 (16)	7 (13)	0.78
Anastomotic leakage	6 (11)	5 (9)	1
Small bowel obstruction and ileus	8 (14)	2 (4)	0.0936
Pneumonia	1 (2)	0 (0)	1
Reoperation	4 (7)	4 (7)	1
Severe postoperative complication	14 (25)	8 (15)	0.23
Hospital stay (days)	25 (17‐35)	16 (12‐24)	0.0014
Preoperative hospital stay (days)	8 (4‐14)	2 (2‐5)	0.0001
Postoperative hospital stay (days)	13 (10‐22)	12 (10‐16)	0.22

Note

After propensity‐score matching.

### 3.4 Multivariate predictors of OS

We performed Cox proportional hazard analysis to determine the predictors of OS (Table 6). The cut‐off value for age was determined using the median. In the multivariate analysis, synchronous distant metastasis (hazard ratio [HR], 1.8; 95% confidence interval [CI], 1.4‐10.9), distant recurrence (HR, 3.6; 95% CI, 1.6‐8.3), and oncologic emergency (HR, 2.4; 95% CI, 1.1‐5.5) significantly indicated a poor prognosis.

**TABLE 6:** Cox proportional hazard analysis for OS

	Univariate analysis			Multivariate analysis		
	HR	95% CI	*P* value	HR	95% CI	*P* value
male	1.3	0.65‐2.77	.43			
age≧70	1.04	0.51‐2.12	.91			
pN+	3.2	1.38‐8.5	.005	1.8	0.7‐5.1	.22
Synchronous distance metastasis	6.89	3.13‐17.8	.0001	3.7	1.4‐10.9	.009
Severe postoperative complications	2.2	0.98‐4.72	.055	2.2	0.89‐5.3	.08
Induction of chemotherapy	1.5	0.73‐3.13	.26			
Distance recurrence	4.4	2.1‐9.4	.001	3.6	1.6‐8.3	.002
Lymph node recurrence	1.66	0.48‐4.3	.37			
Disseminated recurrence	4.2	179‐8.88	.002	2.5	0.9‐6.4	.068
Oncology emergency	2	0.96‐4.4	.064	2.4	1.1‐5.5	.025

Abbreviations: ASA‐PS, American Society of Anesthesiologists physical status classification system.

### 3.5 Subgroup analysis

**FIGURE 2:**
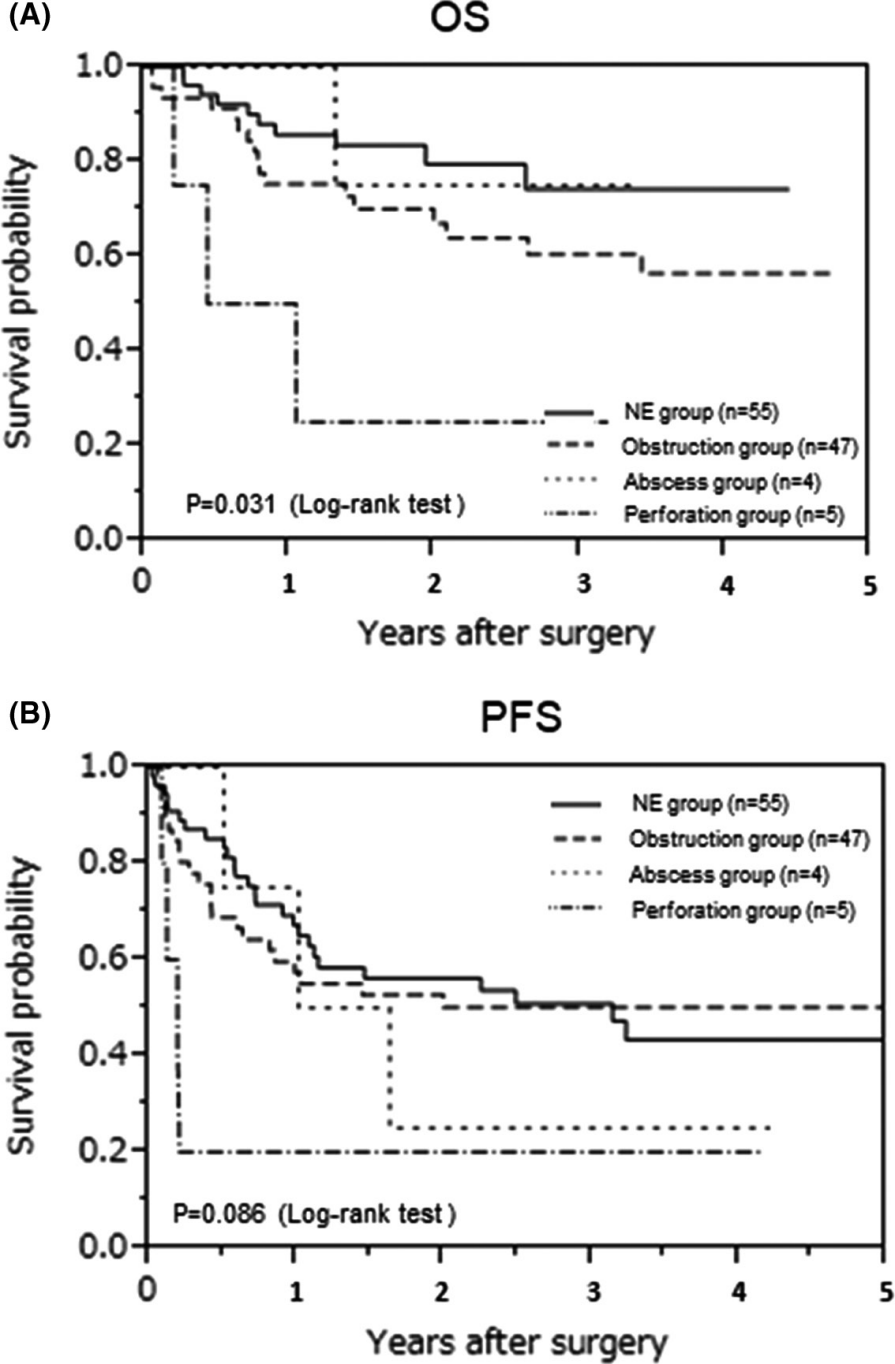
Kaplan‐Meier curve for overall survival and progression‐free survival according to emergency conditions. (A) Overall survival, (B) progression‐free survival

A subgroup analysis was performed to determine whether abscess formation, bowel perforation, or bowel obstruction affected the prognosis. The OS of the perforation group was significantly worse than those of the other three groups (*P* = .003; Figure 2A). Progression‐free survival (PFS) of the abscess and perforation groups showed a trend toward poor prognosis in comparison with the other groups (*P* = .086; Figure 2B). The abscess group showed significantly greater blood loss and significantly more recurrent peritoneal dissemination. The perforation group tended to show a lower rate of chemotherapy induction (Table 7).

**TABLE 7:** Comparison of patients' characteristics and outcomes between the four groups in subgroup analysis

	Abscess group (n = 4)	Perforation group (n = 5)	Obstruction group (n = 47)	NE group (n = 55)	*P* value
《Tumor‐related factor》					
pT3 or pT4	4 (100)	5 (100)	46 (98)	53 (96)	.92
pN+	2 (50)	5 (100)	26 (55)	36 (65)	.21
Synchronous distance metastasis	3 (75)	2 (40)	19 (40)	23 (42)	.6
《Operation‐related factor》					
Operation time (min)	494 (322‐803)	228 (185‐333)	316 (226‐370)	300 (229‐423)	.1
Blood loss (mL)	2840 (765‐10 919)	110 (35‐366)	60 (8‐360)	35 (10‐191)	.012
《Postoperative complications》					
30‐day mortality	0 (0)	0 (0)	2 (4)	0 (0)	.42
Severe postoperative complication	2 (50)	1 (20)	11 (23)	8 (15)	.3
《Long‐term outcomes》					
Induction of chemotherapy	4 (100)	1 (20)	22 (47)	21 (38)	.06
Distance recurrence	1 (25)	2 (40)	16 (34)	19 (34)	.97
Lymph node recurrence	1 (25)	0 (0)	5 (11)	3 (5)	.4
Disseminated recurrence	2 (50)	0 (0)	3 (6)	8 (15)	.045

## 4 DISCUSSION

In this study, we found that CRC patients presenting with an oncology emergency had similar postoperative complications and 30‐day mortality, but worse long‐term prognosis compared with the NE group using propensity‐score matching.

In this study, after adjusting for patient background factors by using propensity‐score matching, there were no significant differences in 30‐day mortality and severe complication rate between the two groups. Surgery could be performed safely even in CRC patients presenting with oncologic emergencies. Lee et al reported that CRC requiring urgent surgery was associated with a significantly higher incidence of postoperative complications and hospital mortality in comparison with the elective surgery group.^5^ Their study showed significant differences in patient background, tumor‐related factors, and surgery‐related factors, suggesting a strong influence of background differences. Because CRC presenting as an oncologic emergency is expected to be a potentially advanced cancer, we adjusted the data for patient background factors, including tumor‐related factors, by using propensity‐score matching in our study. The short‐term outcomes of CRC with oncologic emergencies were similar to those of the waitlist surgery group.

In assessments of long‐term prognosis, the OE group showed a significantly poorer prognosis for OS in the total cohort, and even after adjusting for patient background factors, propensity‐score matching showed that the OE group tended to have a poorer prognosis for OS. The OS of the perforation group was significantly worse than those of the other three groups. Several reports have described the long‐term outcomes of oncologic emergencies in CRC.^6^, ^9^, ^10^, ^11^, ^12^, ^13^ Gunnarsson et al compared the long‐term outcomes in the elective surgery group with those in the oncologic CRC‐related emergency group, which included 87 patients with bowel obstruction and 10 patients with bowel perforation.^6^ Oncologic emergency was reported to be an independent prognostic factor for 5‐year survival (HR 2.25, 95% CI 1.42‐3.55). The prognosis of bowel obstruction in CRC was reported to be worse than that of non‐obstruction because of high local invasion, distant metastasis, and lymph node metastasis.^12^, ^14^, ^15^ Perforation in CRC has been reported to be a poor prognostic factor with the patient facing potential “double jeopardy,” first from the diagnosis of cancer, and second due to the septic complications that accompany perforation.^16^ Tumor perforation was a sign of cancer progression and was reported to promote tumor dissemination, leading to increased recurrence rates and decreased survival.^17^

On the other hand, Martin et al reported that the most common cause of worsening OS in colorectal cancer perforation is perioperative death due to sepsis.^7^ He reported that aggressive source control, oncological resection in hemodynamically stable patients, and the introduction of appropriate postoperative chemotherapy and surgery for recurrence can improve the long‐term prognosis. In their study, PFS and OS seemed to be worsened by the significantly lower postoperative chemotherapy induction rate in the perforation group and the significantly higher postoperative recurrence of peritoneal dissemination in the abscess group.

This study had several limitations. First, this was a single‐center, retrospective study. We used propensity‐score matching to adjust for differences in patient background factors. Since this was a single‐center study with a small number of cases, a multicenter prospective study should be conducted in the future to validate the findings. The second limitation was that the definition of an oncologic emergency was ambiguous. In particular, the bowel obstruction group included mild obstruction that improved with fasting and nasogastric tube placement, which may have improved the short‐ and long‐term prognosis of the OE group. Thus, it seemed necessary to limit the analysis to bowel obstruction requiring decompression procedures (long tube, SEMSs, decompression stoma construction, etc.). The third limitation was that more participants were included in the bowel obstruction group than in the abscess formation and perforation groups. Because of the small number of cases of colorectal cancer perforation at a single institution, a multicenter study is needed. In the future, comparisons limited to bowel obstruction, which is more frequently encountered, and comparisons per decompression method are considered necessary.

## 5 CONCLUSION

Colorectal cancer presenting as an oncologic emergency could be safely treated with surgery without increased perioperative complications in comparison with elective surgery, but the long‐term prognosis was poor.

## DISCLOSURE

Funding: The authors received no funding support for this article.

Approval of the research protocol: The protocol for this retrospective research has been approved by a suitably constituted Ethics Committee of the institution, and it conforms to the provisions of the Declaration of Helsinki. The Ethics Committee of the Graduate School of Medicine, Kumamoto University (Kumamoto, Japan).

Informed Consent: Because this was a retrospective review of medical records, consent to participate was not required from the patients.

Conflict of Interest: Authors declare no conflict of interest for this article.
